# The Role of CTHRC1 in Regulation of Multiple Signaling and Tumor Progression and Metastasis

**DOI:** 10.1155/2020/9578701

**Published:** 2020-08-12

**Authors:** Dan Mei, Yue Zhu, Lingling Zhang, Wei Wei

**Affiliations:** Institute of Clinical Pharmacology, Anhui Medical University, Key Laboratory of Anti-inflammatory and Immune Medicine, Ministry of Education, Anhui Collaborative Innovation Center of Anti-inflammatory and Immune Medicine, Hefei, 230032 Anhui Province, China

## Abstract

Collagen triple helix repeat containing-1 (CTHRC1) has been identified as cancer-related protein. CTHRC1 expresses mainly in adventitial fibroblasts and neointimal smooth muscle cells of balloon-injured vessels and promotes cell migration and tissue repair in response to injury. CTHRC1 plays a pivotal role in some pathophysiological processes, including increasing bone mass, preventing myelination, and reversing collagen synthesis in many tumor cells. The ascended expression of CTHRC1 is related to tumorigenesis, proliferation, invasion, and metastasis in various human malignancies, including gastric cancer, pancreatic cancer, hepatocellular carcinoma, keloid, breast cancer, colorectal cancer, epithelial ovarian cancer, esophageal squamous cell carcinoma, cervical cancer, non-small-cell lung carcinoma, and melanoma. And molecules that regulate the expression of CTHRC1 include miRNAs, lncRNAs, WAIF1, and DPAGT1. Many reports have pointed that CTHRC1 could exert different effects through several signaling pathways such as TGF-*β*, Wnt, integrin *β*/FAK, Src/FAK, MEK/ERK, PI3K/AKT/ERK, HIF-1*α*, and PKC-*δ*/ERK signaling pathways. As a participant in tissue remodeling or immune response, CTHRC1 may promote early-stage cancer. Several recent studies have identified CTHRC1 as an effectual prognostic biomarker for predicting tumor recurrence or metastasis. It is worth noting that CTHRC1 has different cellular localization and mechanisms of action in different cells and different microenvironments. In this article, we focus on the advances in the signaling pathways mediated by CTHRC1 in tumors.

## 1. Introduction

Collagen triple helix repeat containing-1 (CTHRC1), an extracellular matrix (ECM) protein, was identified in the screening of differentially expressed sequences between balloon injury and normal arteries. The evolution of Cthrc1 can be traced back to at least 550 million years ago, and the conserved genes were not found in invertebrates [1]. CTHRC1 has complicated interactions with various intracellular and extracellular matrices in different ways of secretion [2, 3]. CTHRC1 increases the activity of collagen promoter through binding to ligands and could contribute to vascular remodeling by limiting collagen matrix deposition and promoting cell migration [4]. CTHRC1 promotes the recruitment of M2 macrophages and regulates TGF-*β* and Notch pathways to accelerate wound healing in a mouse model of acute wound healing [5]. As a coupling factor, CTHRC1 can be secreted by osteoclasts and influence bone formation and remodeling by acting on osteoblasts and osteocytes [6, 7]. CTHRC1 may promote IL-1*β*-induced apoptosis of chondrocytes by activating the JNK1/2 pathway [8]. The anti-inflammatory effect of CTHRC1 expressed on activated synovial cells was also found in a collagen antibody-induced arthritis model [9]. Besides, CTHRC1 can regulate physiological functions such as fat and glycogen synthesis and promote autonomous activity [3, 10].

Therefore, as a secreted protein, CTHRC1 is involved in multiple pathophysiologies. A remarkable effect is that the high expression of CTHRC1 promotes tumorigenesis and development through positive regulation of tumor spread, invasion, migration, adhesion, and metastasis. CTHRC1 exerts its effects through several signaling pathways such as TGF-*β*, Wnt, integrin *β*/FAK, Src/FAK, MEK/ERK, PI3K/AKT/ERK, HIF-1*α*, and PKC-*δ*/ERK signaling pathways. In this article, we focus on the advances in the signaling pathways mediated by CTHRC1 in tumors.

## 2. The Structural Characteristics and Expression of CTHRC1

### 2.1. The Structural Features of CTHRC1

The CTHRC1 gene is located at chromosome 8q22.3, and it contains five exons in humans and four exons in mice. It covers 11.50 kb on the direct strand and can be transcribed into 1.2 kb mRNA. The amino acid sequence identity between human and rat CTHRC1 proteins was 92%, and no homologs were found in lower species [10, 11].

**Figure 1:**
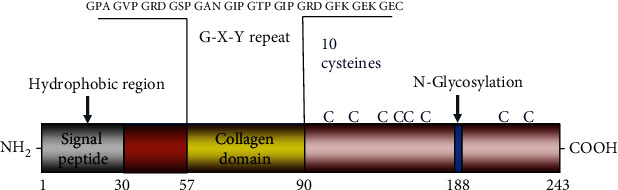
The structure of the CTHRC1 protein. The construct of CTHRC1 contains an NH2-terminal peptide for extracellular secretion, a short collagen triple helix repeat of 36 amino acids, and a COOH-terminal globular domain. The proline-rich hydrophobic domain lies between the 1st and 30th amino acids and serves as a signal peptide for transport to the endoplasmic reticulum. CTHRC1 comprises a collagen domain between amino acids 57 and 90, and the protein contains 10 cysteine residues, corresponding to about 4.7% cysteine in the final protein. What is more, its only amino acid posttranslational modification is the glycosylation of asparagine at position 188.

Secreted CTHRC1 exists primarily as a dimer (56 kDa) and a trimer (84 kDa) as well as multimers of the trimeric CTHRC1 (168 kDa and 252 kDa). The construct of CTHRC1 contains an NH2-terminal peptide for extracellular secretion, a short collagen triple helix repeat of 36 amino acids, and a COOH-terminal globular domain [1, 12]. Similar molecular weight and structural characteristics to adiponectin also explain why CTHRC1 can form high molecular weight complexes [13]. The biological activity of CTHRC1 is restricted to the highly conserved 200 amino acids at the C-terminal region, and the C-terminal region of CTHRC1 contains a putative N-glycosylation site that stabilizes CTHRC1 protein. N-Glycosylation also promotes CTHRC1 to tether to the cell membrane, which promotes actin polymerization and cell polarity [14]. A short collagen motif with 12 Gly-X-Y repeats presents in C1q/tumor necrosis factor-*α*-related proteins (CTRPs), which appears to be responsible for the trimerization of protein and renders molecule susceptible to cleavage by collagenase (see Figure 1). However, dimeric CTHRC1 would not be susceptible to cleavage by collagenase [1, 15]. The molecular weight of secreted CTHRC1 (30 kDa) appears to be larger than that of cellular CTHRC1 (26 kDa). CTHRC1 has four different isoforms with molecular weights of about 12.3 kDa to 27 kDa; the full-length of CTHRC1 accounts for both secreted and cellular CTHRC1 [12]. Glycosylated protein CTHRC1 with a signal sequence is related to ECM and contains a variable short collagen-like motif. Intriguingly, CTHRC1 plays a role in inhibiting structural proteins, unlike other members of the collagen family [1, 16]. Moreover, Leclair et al. found that Cthrc1 cleaved at the N-terminus by plasmin shows better inhibition of collagen synthesis compared to full-length Cthrc1 in the PAC1 cell line [17]. These studies suggested that CTHRC1 might obtain biological activity through proteolytic processing.

### 2.2. The Expression of CTHRC1

CTHRC1 is transiently expressed by fibroblasts in remodeling adventitia and by smooth muscle cells in the neointima of injured tissue; however, CTHRC1 is not detected in normal arteries. In injured arteries and skin, the expression of CTHRC1 is associated with myofibroblasts and locates in the sites of collagen matrix deposition. In mice, the first exon of CTHRC1 was targeted to be replaced with a *β*-galactosidase expression construct, which demonstrated the expression of CTHRC1 in inner ear hair cells [14]. There is CTHRC1 expression in many mesenchymal-derived cells during body growth and tissue repair [10]. In mouse embryos, CTHRC1 is expressed in visceral endoderm, notochord, neural tube, developing kidney, and heart. Abundant expression of CTHRC1 is observed in developing skeleton, including cartilage primordia, growth plate cartilage, bone matrix, and periosteum. In adults, CTHRC1 is expressed only in bone matrix and periosteum. CTHRC1 is also found in the matrix of calcifying atherosclerotic plaques and mineralized bone of skeletal tissues in humans. In other tissues, the sites of CTHRC1 expression overlap considerably with interstitial collagens and transforming growth factor-*β* (TGF-*β*) family members, particularly bone morphogenetic proteins (BMPs). The sites of CTHRC1 expression are characterized by the presence of active TGF-*β* and abundant collagen synthesis [18]. CTHRC1 mRNA expression levels are increased in response to BMP-4, BMP-2, and TGF-*β*. Furthermore, TGF-*β* signaling could lead to a significant increase in neointimal lesion formation [19, 20]. The expression of CTHRC1 is also positively correlated with tumor lymph node metastasis, tumor-node-metastasis (TNM) stage, and disease prognosis. However, its potential regulatory mechanisms in the tumor environment have not yet been elucidated.

## 3. The Molecules That Regulate the Expression of CTHRC1

CTHRC1 is abnormally expressed in several solid tumors, especially in gastric cancer, pancreatic cancer, hepatocellular carcinoma, keloid, breast cancer, colorectal cancer (CRC), epithelial ovarian cancer, esophageal squamous cell carcinoma (ESCC), cervical cancer, non-small-cell lung carcinoma (NSCLC), melanoma, and so on [11, 21–31]. And molecules that regulate the expression of CTHRC1 include miRNAs, lncRNAs, WAIF1, and DPAGT1.

### 3.1. miRNAs

Microribonucleic acids (miRNAs), which can regulate gene expression, are a class of noncoding single-stranded small RNAs of about 22 nucleotides that can inhibit the mRNA translation process by exclusively promoting the degradation of several mRNAs [32]. In many tumors, miRNAs, such as miR-30c, miR-9, miR-520d-5p, miR-155-5p, miR-98, Let-7b, miR-155, miR-101, and miR-217 can regulate the expression of CTHRC1.

miR-30c could regulate CTHRC1 at a posttranscriptional level in breast cancer. It downregulates the CTHRC1-mediated GSK-3*β*/*β*-catenin signal and inhibits tumor cell proliferation, invasion, and migration. In addition, miR-30c can also upregulate Bax/Caspase-9/Caspase-3, a downstream signal of CTHRC1 inhibiting apoptosis [33]. In hepatocellular carcinoma, CTHRC1 downregulates miR-155-5p through the activation of GSK-3*β*-involved Wnt/*β*-catenin signaling to promote tumor formation [34]. And miR-98 dramatically downregulates CTHRC1 by directly targeting the 3′-UTR of CTHRC1 suppressing hepatocellular carcinoma formation [35]. miR-9 could inhibit the migration of Schwann cell by targeting CTHRC1 following sciatic nerve injury, thereby inactivating downstream Rac1 GTPase [36]. miR-520d-5p is significantly downexpressed and suppresses cell proliferation, migration, and invasion by targeting CTHRC1 in CRC [37]. As the second miRNA following lin-4 in Caenorhabditis elegans, Let-7b may directly target CTHRC1 and function as a tumor suppressor gene in gastric cancer [38, 39]. In ESCC, miR-101 and miR-217 could inhibit the expression of CTHRC1 [40]. miR-30 could downregulate the expression of Cthrc1 and downstream signal molecules such as MMP-9 and MMP-2 to inhibit the invasion and migration of NSCLC cells [41]. A recent study found that miR-155 downregulation and CTHRC1 upregulation were observed in CRC. Moreover, overexpression of miR-155 can silence downstream CTHRC1, thereby inhibiting cell proliferation and inducing apoptosis of cells to prevent tumor progression and metastasis [42]. In conclusion, the negative regulation of CTHRC1 by miRNA has the potential to become a novel direction for cancer treatment in the future.

### 3.2. lncRNAs

Metastasis-associated lung adenocarcinoma transcript I (MALAT-1) is a large, infrequently spliced long noncoding RNA (lncRNA), which could genetically increase CTHRC1 activity to regulate lung cancer cell migration [43]. The silence of MALAT-1 could also inhibit the expression of CTHRC1 which is a positive regulator of ESCC [40]. Further, another lncRNA named NONMMUG014387 could also regulate CTHRC1 and activate the Wnt/PCP pathway to promote Schwann cell proliferation at the site of injury [44].

### 3.3. WAIF1

Wnt-activated inhibitory factor 1 (WAIF1) is silenced by promoter hypermethylation in various cancers [45, 46]. LC-MS/MS analysis (using liquid chromatography and mass spectrometry analysis of samples) of CTHRC1-binding membrane proteins indicates that the largest part of CTHRC1 binds the WAIF1 receptor [47]. Recent research suggests that WAIFI expression is activated by suppressing methylation of its promoter. Activated WAIF1 downregulates the expression of Wnt/*β*-catenin target genes to inhibit the development of endometrial adenocarcinoma [48]. The binding of CTHRC1 to WAIF1 could promote osteoblast differentiation [49]. Therefore, CTHRC1-WAIF1 interaction can be a potential therapeutic target in the future.

### 3.4. DPAGT1

N-Glycosylation is essential for the migration pattern of immune cells, and its dysregulation is related to various diseases including cancer. In human ESCC, the overexpression of CTHRC1 is associated with hyperglycosylation and promoter hypomethylation [50]. Increased N-glycosylation is associated with preferential localization of CTHRC1 in wound cells. And N-glycosylation facilitates the promigratory function of CTHRC1. Dolichyl-phosphate N-acetylglucosamine-phosphotransferase 1 (DPAGT1), the gene that encodes the first enzyme and rate-limiting enzyme in the assembly of lipid-linked oligosaccharide precursors in the endoplasmic reticulum, is related to the formation of mature intercellular adhesion complexes [51]. As an upstream regulator of N-glycosylation status of E-cadherin, DPAGT1 could upregulate CTHRC1 by increasing protein turnover, indicating that N-glycosylation can also stabilize CTHRC1 [52].

Besides, TGF-*β* and FAK could also regulate the expression of cthrc1 in different signaling pathways. It should be highlighted that CTHRC1 not only is the result of tumor progression but also plays a predominant regulatory role in the progression and metastasis of many solid tumors [2, 53–55].

In summary, many molecules can regulate the expression and activity of CTHRC1 and together with CTHRC1 as novel antitumor molecular targets for the treatment of cancer in the future.

## 4. Signaling Pathways Mediated by CTHRC1 Involved in the Progression and Metastasis of Tumor

**Figure 2:**
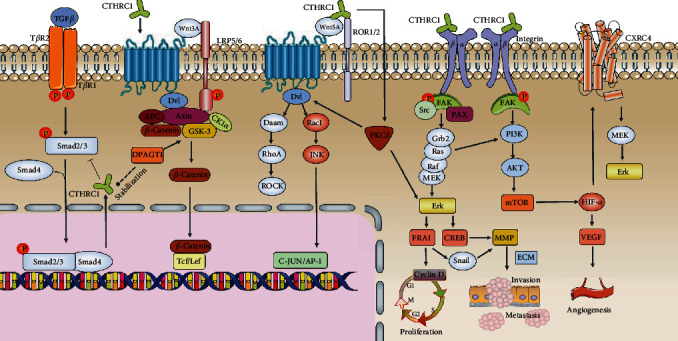
Signaling pathways mediated by CTHRC1 involved in the progression and metastasis of tumor. (1) TGF-*β* signaling pathway is quite complex, especially in terms of its effects, which are often contradictory depending on location and time. There exists a critical negative feedback regulatory loop between TGF-*β*-smad2/3 signaling pathway and CTHRC1. (2) WNT signaling includes WNT/*β*-catenin canonical pathway and *β*-catenin-independent noncanonical pathway. In the canonical WNT signaling, Fzd receptor and LRP5/LRP6 coreceptor are transduced to *β*-catenin signaling cascade for the maintenance of stem and progenitor cells. In the noncanonical WNT signaling, Fzd receptor and ROR2/PTK7/RYK coreceptor are transduced to RhoA, JNK signaling cascades for the control of tissue polarity, cell adhesion, or cell movement. The downstream molecules of the WNT/PCP pathway mainly include the small GTPase family, such as Rac1, RhoA, and JNK, which play essential roles in cancer cell migration and invasion. (3) CTHRC1 signal via WAIF1 can activate PKC*δ*, which is an essential component of the WNT/PCP pathway. Furthermore, PKC*δ* is responsible for the activation of the CTHRC1-induced ERK signaling pathway. (4) In CTHRC1/integrin *β* signaling pathway, the upregulation of CTHRC1 is related to the progression and metastasis of several cancers through the activation of several key signaling molecules, including Src, FAK, paxillin, MEK, ERK, and Rac1. FAK promotes cancer cell migration by regulating focal adhesion formation and turnover, which involve activation of Src and paxillin. FRA-1 is activated by CTHRC1 through the MAPK/MEK/ERK signaling, which leads to the upregulation of cyclin D1 and that promotes cell proliferation. FRA-1 also induces snail1-mediated MMP14 expression to facilitate ESCC cell invasion, migration, and metastasis. PI3K/AKT signaling pathway induces EMT change and MMP2/MMP9 expression. (5) HIF-1*α* and VEGF are activated by CTHRC1 through activating the PI3K/AKT/mTOR signaling pathway, which promotes tumor angiogenesis. CTHRC1 also participates in tumor cell migration and invasion through HIF-1*α*/CXCR4 signals.

The influence of CTHRC1 on various events in tumor progression is based on its regulation of various signaling pathways such as TGF-*β*, Wnt, integrin *β*/FAK, Src/FAK, MEK/ERK, PI3K/AKT/ERK, HIF-1*α*, and PKC-*δ*/ERK signaling pathways (see Figure 2). These properties' pathways affected by CTHRC1 play an essential role not only in tissue remodeling after injury, regulation of ossification, and other physiological processes but also in the development of cancer and metastasis.

### 4.1. Negative Feedback Regulation of CTHRC1 and Cell Type-Specific TGF-*β* Signaling Pathway

As the most potent growth factor involved in wound healing, TGF-*β* is released by platelets at the site of injury, influencing inflammatory response, angiogenesis, reepithelialization, ECM, and remodeling [56]. TGF-*β* superfamily members include TGF-*β*, activin, and BMPs. Smad1/5/8 mediates BMP signaling, while smad2/3 mediates TGF-*β* and activin signaling.

CTHRC1 has been reported to have a relationship with the TGF-*β* family since its discovery, as their expression sites overlap significantly. TGF-*β*1 and BMP4 can induce the transcription and expression of CTHRC1 in NIH3T3 cells [1]. CTHRC1 can activate TGF-*β* signaling via an elevation in Smad2/Smad3 phosphorylation. Activated Smad2/3 forms a complex with Smad4 and accumulates in the nucleus, causing an increase in collagen type I deposition during vascular remodeling [57–59]. There exists a critical negative feedback regulatory loop between TGF-*β*1 and CTHRC1. The conserved region of 90-119 amino acids in CTHRC1 protein can bind to phospho-Smad3. CTHRC1 is induced by TGF-*β*1 via phospho-Smad3 binding to the promoter with subsequent transcription activation. And in turn, CTHRC1 inhibits TGF-*β*1 signaling by accelerating proteasomal degradation of phospho-Smad3, which inhibits collagen deposition. TGF-*β* can enhance the migration and invasion characteristics of endothelial cells by regulating the secretion and expression of MMP-2 and MMP-9 [60]. Therefore, inhibiting CTHRC1-mediated TGF-*β* signaling pathways may effectively suppress the invasion and angiogenesis of cancer cells [12, 61].

However, the mechanism of TGF-*β* involved in tumor progression is very complex. Even in the same tumor type, TGF-*β* has many different roles in tumor progression. For example, the activation of nuclear factor of activated T-cells (NFATs) can drive the switch of the tumor-suppressive function of TGF-*β* towards tumor progression [61, 62]. TGF-*β* increases the level of CTHRC1 in CRC cells. Highly expressed CTHRC1 promotes epithelial-mesenchymal transition (EMT) and tumor metastasis through the Smad2/Smad3 activation of TGF-*β* pathway [63]. CTHRC1 can also inhibit the TGF-*β*/Smad pathway and YAP nuclear translocation, thereby inhibiting type I collagen synthesis [64]. Metabolites such as bile acid may induce CTHRC1 to activate the TGF-*β*-smad2/smad3 pathway to mediate liver fibrosis and may progress towards hepatocellular carcinoma [65, 66]. In the polyvinyl alcohol sponge model, CTHRC1 activates TGF-*β* and Notch pathways to promote the recruitment of M2 macrophages. However, CTHRC1 may downregulate TGF-*β* expression during the late remodeling phase of wound healing [5]. TGF-*β* regulates the expression of CTHRC1 in a concentration-dependent manner in keloids, and excess CTHRC1 reverses collagen synthesis [30]. Therefore, these results of the regulation between CTHRC1 and TGF-*β* are not contradictory. Other than that, CTHRC1 has no inhibitory effect on TGF-*β* signaling in endothelial cells [67]. These results indicate that the regulation of TGF-*β* by CTHRC1 may play a role in other interstitial cells of the tumor microenvironment and that this regulation is cell type-specific. The further exploration of detailed molecular mechanism by which CTHRC1 activates the TGF-*β* pathway may resolve these disputes.

### 4.2. Mutual Regulation between CTHRC1 and Wnt Pathways to Promote Tumor Progression and Metastasis

Wnt family are secreted glycoproteins; include Wnt-1, Wnt-1, Wnt-3a, Wnt-4, Wnt-5a, Wnt-5b, Wnt-6, Wnt-7a, and Wnt-7b; and participate in the process of numerous oncogenic and development progress [68–70]. Wnt5a is a member of the Wnt protein family and plays an essential role in the pathological process of neuropathy and malignant tumors [70–72]. Wnt proteins activate the Wnt/*β*-catenin canonical pathway and *β*-catenin-independent noncanonical pathway, among which the planar cell polarity (PCP) pathway and Wnt/calcium (Ca^2+^) pathway are the most widely studied [73–75]. Current reports indicate that CTHRC1 is mainly involved in tumor progression through the canonical Wnt/*β*-catenin and noncanonical Wnt/PCP pathways.

#### 4.2.1. Wnt/*β*-Catenin Canonical Signaling Pathway

In the Wnt/*β*-catenin canonical pathway, Wnt proteins bind to Frizzled (Fzd) receptor and lipoprotein receptor-related protein 5/6 (LRP5/6) coreceptor. In the absence of Wnt signaling, the cytoplasmic *β*-catenin form the “destruction complex” composed with the casein kinase 1*α* (CK1*α*), glycogen synthase kinase 3*β* (GSK3*β*), adenomatous polyposis coli (APC), and Axin, which activates the EMT to promote cancer invasion and metastases through CTHRC1/Wnt/*β*-catenin [76, 77]. The level of *β*-catenin is maintained as low by the series of events, including priming phosphorylation by CK1*α* at Ser45 and subsequently at Thr41, Ser37, and Ser33 by GSK3*β* [78, 79]. When secreted Wnt ligands are accumulated, Wnt combines with Fzd receptor, and LRP5/6 coreceptors lead to activation of dishevelled (DVL) protein [80]. The activated DVL is phosphorylated and translocated to the Fzd receptor [81], causing the dissociation of the *β*-catenin “destruction complex” and the cytosolic accumulation of *β*-catenin. As the cytosolic *β*-catenin accumulates, RAS proteins are accumulated due to the absence of GSK3*β*-mediated phosphorylation. The stabilized RAS proteins at the plasma membrane activate RAF/mitogen-activated protein kinase (MEK)/extracellular signal-regulated kinase (ERK) cascade [82]. Besides, cytosolic *β*-catenin subsequently translocates into the nucleus and forms a complex with the T-cell factor (TCF) or lymphoid enhancer factor (LEF). The complex activates the expression of target genes involving proliferation and transformation, such as c-MYC, c-Jun, CCND1 (gene encoding cyclin D1), epidermal growth factor receptor (EGFR), CD44, CD133, and leucine-rich repeat-containing G protein-coupled receptor 5 (LGR5) [83–87].

The Wnt/*β*-catenin signaling pathway plays an indispensable role in the occurrence and development of many types of cancer. CTHRC1-induced nuclear translocation of *β*-catenin was observed in NCL-H23 cells, and luciferase assay showed that *β*-catenin/TCF transcriptional activity was enhanced. In contrast, the knockdown of CTHRC1 reduced the *β*-catenin/TCF transcriptional activity, which shows that CTHRC1 regulates the invasiveness of NSCLC cells through the Wnt/*β*-catenin pathway [27]. Similarly, CTHRC1 activates snail1 through the Wnt/*β*-catenin signaling pathway to promote EMT in epithelial ovarian cancer [88]. During the development and metastasis of CRC, CTHRC1 may promote the activation of the Wnt signaling pathway through ANOS1 [89]. It can also participate in the Wnt/*β*-catenin pathway to regulate the malignant behavior of hepatocellular carcinoma with GSK-3*β* [34]. Many cancers usually metastasize to bone in advanced stages. CTHRC1 secreted by osteoclasts promotes basic fibroblast growth factor (bFGF) expression in osteoblasts by stimulating Wnt/*β*-catenin signaling, which may induce the development of cancerous bone lesions but not mediate vascular production [90]. The constitutive activation of the Wnt-*β*-catenin pathway leads to carcinogenesis in tumors [91]. CTHRC1 promotes *β*-catenin nuclear translocation and induces transcription of downstream target genes (such as c-Myc and cyclin D1) in the nucleus, reduces cell adhesion, and promotes cell proliferation [92]. Subsequently, tumor cell invasion and metastasis occurred.

Interestingly, another article reported that *β*-catenin could act on the CTHRC1 promoter region and promote transcription. N-Glycosylation stabilizes CTHRC1 in oral squamous cell carcinoma (OSCC) specimens by reducing protein turnover rate, and CTHRC1 is positively feedback-regulated by the DPAGT1/canonical Wnt pathway, thereby activating noncanonical Wnt pathways to drive tumor cell migration and invasion [52]. In contrast, the overexpression of CTHRC1 in HEK293T cell and gastrointestinal stromal tumor (GIST) cell significantly inhibited the canonical Wnt pathway but activated the noncanonical Wnt/PCP pathway [14, 93]. Based on the evidence reviewed above, it can be indicated that crosstalk between the canonical Wnt/*β*-catenin pathway and noncanonical Wnt/PCP pathway and the mutual regulation of Wnt/*β*-catenin and CTHRC1 accelerate the process of tumor progression.

#### 4.2.2. Wnt/PCP Noncanonical Signaling Pathway

Early reports suggest that CTHRC1 activates the PCP pathway during inner ear development [14]. CTHRC1 can interact with multiple extracellular components of WNT signaling, Fzd proteins, and Wnt/PCP coreceptor ROR2. The components form a CTHRC1-Wnt-Fzd/ROR2 complex to activate the Wnt/PCP pathway selectively and transmit signals from the cell-surface complex to the nucleus by Dvl-RhoA/Rac1-JNK-ATF2/c-Jun cascade, promoting cancer cell protrusions, proliferation, migration, and invasion [14, 76, 93–98]. CTHRC1 is capable of coordinating three small Rho GTPases (Rac1, RhoA, and Cdc42), which are the leading performers of Wnt/PCP to promote cell migration. In cervical cancer, CTHRC1 cooperates with E6/E7 human papillomavirus (HPV) to activate the noncanonical Wnt/PCP pathway, which aggravates tumor malignancy [2]. In pancreatic cancer and human urothelial carcinoma, Wnt5a/ROR2 signaling is associated with EMT and promotes tumor cell invasion and metastasis [99, 100]. In GIST, CTHRC1 appears to activate the Wnt/PCP pathway in a dose-dependent manner, and Wnt5a/PCP-Rho axis determines the tumor invasion-promoting activity of CTHRC1 [93]. A recent study demonstrated that CTHRC1 could promote ERK and JNK phosphorylation by activating PCP signaling pathways in human umbilical vein endothelial cells (HUVECs) and promote tumor angiogenesis [101]. What is more, it was observed that the paracrine CTHRC1 controls the expression of Ang-2 via noncanonical Wnt pathway activation of ERK-dependent AP-1 in HUVECs [54]. Hence, over and above that associated with the canonical Wnt/*β*-catenin pathway, noncanonical Wnt signaling pathways interact with other signaling pathways.

### 4.3. CTHRC1 Binds Integrin *β* and Triggers a Series of Signaling Cascades to Promote Tumor Progression and Metastasis

#### 4.3.1. Integrin *β*/FAK/Src Signaling Pathway

Integrins are transmembrane receptors that promote cell-ECM adhesion. With two subtypes of *α* and *β*, it can participate in a variety of physiological activities such as tumor progression and migration [102]. CTHRC1 promotes hepatocellular carcinoma cell invasion by activating the RhoA/Rho-associated kinase (ROCK) pathway and facilitates adhesion of hepatocellular carcinoma cells to ECM through induction of integrin *β*1 expression and activation of focal adhesion kinase (FAK) [103]. Another study of hepatocellular carcinoma suggests that CTHRC1 inhibits anoikis and increases tumor cell survival by activating integrin *β* expression [104]. Cell adhesion to fibronectin is mediated by integrin *β*1 [105]. Previous researches have demonstrated that targeting the integrin *β*3/FAK signaling could enhance the antitumor activity and attenuate cancer metastasis, including melanoma, endometrial cancer, NSCLC, and ESCC [106–111]. Guo et al. found that phosphorylated FAK was significantly reduced in mice with EOC xenograft tumors, and inhibition of FAK did not interfere with integrin *β*3 expression in vivo. However, the overexpression of CTHRC1 leads to the upregulation of integrin *β*3 in model mice, proving that CTHRC1 interacts with integrin *β*3 and promotes FAK phosphorylation at Tyr397, thereby promoting ovarian cancer cell adhesion, migration, and invasion [112].

The high level of CTHRC1 is connected with the progression and metastasis of pancreatic cancers through the activation of several key signaling molecules, including the steroid receptor coactivator (Src), FAK, paxillin, MEK, ERK, and Ras-related C3 botulinum toxin substrate 1 (Rac1) [77]. Src/FAK signaling cascade takes a regulative role in regulating the formation of protein complexes at focal adhesions in the migration and metastasis of cancer cells [113]. Src can correspond to integrin-ECM interaction and is recruited to form the Src/FAK complex, which permits FAK to be active [114, 115]. Then, FAK activates Src and paxillin by regulating focal adhesion formation and turnover [116]. Paxillin, a focal-adhesion adaptor molecule, serves as a scaffold for the organization and the activation of Raf, MEK, and ERK [117, 118]. Furthermore, paxillin can stimulate Rac1, which is a Ras superfamily member of small guanosine triphosphatase (GTPase) and a critical factor in cytoskeleton reorganization, regulation of gene expression and cell proliferation, and cellular transformation [119–121].

ERK2-mediated paxillin phosphorylation promotes FAK adhesion to focal adhesions [122]. Additionally, the inhibition of FAK-paxillin interaction results in decreased phosphorylation of FAK and its targets, which in turn changes cell adhesion and migration. This evidence has inspired the development of anticancer drugs targeting FAK [123]. FAK-Src complex plays essential functions in TGF-*β*-induced hepatocyte EMT models, such as upregulating MMP9 and fibronectin and downregulating E-cadherin [124].

#### 4.3.2. MEK/ERK and PI3K/AKT/ERK Signaling Pathway

CTHRC1 interacts with integrin to trigger a series of signal cascades. Because Src can phosphorylate other FAK sites, it can recruit proteins containing Src homology 2 (SH2) domains such as Grb2. Subsequently, the downstream Ras-MAPK pathway and the phosphatidylinositol 3- kinase- (PI3K-) AKT cascades are activated to participate in cellular response [125]. CTHRC1 activates Fos-related antigen-1 (FRA-1) through MAPK/MEK/ERK signaling, which leads to the upregulation of cyclin D1, and promotes cell proliferation. FRA-1, a FOS family transcription factor, also induces snail1-mediated MMP14 expression to facilitate ESCC cell invasion, migration, and metastasis [50]. Snail1 transcriptional factor is essential for triggering EMT and inducing tumor cell invasion [126]. Knocking down CTHRC1 will change the phosphorylation level of ERK1/2 and thus regulate the pathological process of endometriosis (EM) [127]. The frequent upregulation of CTHRC1 observed in human colon cancer cells may be due to a CpG demethylation event in the exon 1 region of the gene. Kim et al. tested the luciferase reporter gene using the ERK-responsive ELK1 promoter, proposing that CTHRC1 upregulates MMP9 through ERK activation. Further, treatment with MEK1/2 inhibitors can reduce tumor cell invasion, and ERK activation and aggressiveness are reduced by knocking down CTHRC1 [23].

CTHRC1 promotes invasiveness and metastasis of hepatocellular carcinoma through the activation of PI3K/protein kinase B (PKB)/AKT/ERK/cAMP response element-binding protein (CREB) signaling pathway, which induces EMT change and MMP2/MMP9 expression [128]. CTHRC1 is highly expressed in hepatitis B virus- (HBV-) associated hepatocellular carcinoma. HBV activates nuclear factor-kappa B (NF-*κ*B) and CREB through the ERK/c-Jun N-terminal kinase (c-JNK) pathway to stimulate CTHRC1 expression. In addition, hypoxia-inducible factor 1*α* (HIF-1*α*) and vascular endothelial growth factor (VEGF) are activated by CTHRC1 through activating the PI3K/AKT/mammalian target of rapamycin (mTOR) signaling pathway, which promotes tumor angiogenesis. What is more, CTHRC1 enhances colony formation, migration, and invasion of hepatocellular carcinoma cells by downregulating tumor suppressor (p53) and stimulating invasion-associated factor (MMP-9) [129].

Studies on myocardial infarction (MI) have also found that activation of infarct repair cardiac fibroblasts (IRCF) involves CTHRC1 expression and PI3K-Akt signaling pathway. In ovarian cancer cells, gene silencing CTHRC1 does not alter MMP9 expression or phosphorylate MEK. The invasion-promoting effect of CTHRC1 on EOC cells depends on downstream PI3K/AKT and ERK1/2 signaling dominated by EGFR [130]. Besides, the invasion and metastasis of endometrial cancer are closely related to the upregulation of CTHRC1-mediated CX3CR1 in macrophages. This process regulates the integrin *β*3/PI3K/AKT pathway, which also promotes the recruitment of M2-like macrophages [55].

### 4.4. CTHRC1 Activates HIF-*α* Pathway and Contributes to Tumor Angiogenesis

As mentioned above, CTHRC1 in hepatocellular carcinoma can induce HIF-1*α* to promote tumor angiogenesis and regulate downstream MMPs and tumor suppressor gene p53 by activating the PI3K/AKT signaling pathway [128]. In human squamous cell carcinoma, HIF-1*α* overexpression stimulates VEGF-C upregulation and induces lymphangiogenesis and tumor cell invasion [131]. Ding et al. pinpointed that CTHRC1 and HIF-1*α* were upregulated in the nucleus of CTHRC1 overexpressed GC cells. HIF-1*α* inhibitors reduced CTHRC1-induced CXCR4 expression. Furthermore, it was found that inhibition of HIF-1*α* expression and inhibition of CXCL12/CXCR4 signals all alleviate tumor cell migration and invasion. Therefore, CTHRC1 can participate in tumor cell migration and invasion through HIF-1*α*/CXCR4 signals in GC [132]. In short, CTHRC1 can affect the expression of HIF-1*α*, which is not only related to lymphangiogenesis but also closely related to tumor progression and invasion.

### 4.5. A Novel Signaling Pathway: The Potential Role of PKC-*δ*/ERK in Tumors

Protein kinase C *δ* (PKC-*δ*) has been implicated in various epithelial tumors such as prostate, breast, and CRC [133, 134]. Activated PKC-*δ* causes angiogenesis and tumor growth of prostate tumors by increasing NADPH oxidase activity and HIF-1*α* expression levels [135]. PKC-*δ* can also inhibit the Wnt/*β*-catenin pathway in colon cancer cells [136]. However, a recent study illustrated that MEK and PKC-*δ* inhibitors could block CTHRC1-induced ERK phosphorylation and that PKC-*δ* phosphorylation was not inhibited by MEK inhibition. Surprisingly, inhibition of PLC, a membrane-associated enzyme that activates PKC-*δ* to promote bone formation in noncanonical Wnt signals, did not inhibit CTHRC1-induced alkaline phosphatase (ALP) activity. Therefore, WAIF1 bound by CTHRC1 activates PKC-*δ* and ERK to stimulate osteoblast differentiation, which is a novel signaling pathway unrelated to the noncanonical Wnt pathway [49]. Therefore, PKC-*δ* signal may explain the role of CTHRC1 in tumor progressions such as angiogenesis and bone metastasis.

To put it briefly, CTHRC1 may be involved in many other signaling pathways (including miRNA and LncRNA), which interact with or crosstalk with the TGF-*β*, Wnt, and integrin *β*/ERK pathways, and jointly participate in tumor development and metastasis (see Table 1).

## 5. Conclusion

Tumor development and metastasis, a complex process involving cell adhesion and proteolytic degradation of the ECM, depends not only on the cancer cells but also on the interaction between the cancer cells and their microenvironment. Complementary DNA microarray analysis also demonstrated that the CTHRC1 gene is expressed in most human solid cancers. As we all know, CTHRC1 is a secreted ECM protein, which can inhibit collagen deposition and participate in tumor invasion and metastasis. Even though CTHRC1 was first discovered more than a decade ago, research into its related signaling pathways and functions has never been interrupted and is still providing surprising findings. The role of CTHRC1 in promoting tumor angiogenesis can serve as a research marker for the pathogenesis of autoimmune diseases such as rheumatoid arthritis. As a participant in tissue remodeling or immune response in the tumor microenvironment, CTHRC1 may also participate in early-stage cancer. Inhibiting CTHRC1 can isolate cancer cells from spreading to nearby organs.

In addition to the targeted regulation of CTHRC1 by miRNAs, it has been demonstrated that cyclovirobuxine-D could inhibit the progression and metastasis of CRC cells due to the targeting of CTHRC1 [137]. Therefore, CTHRC1 may be a potential target in combination with antitumor drugs in the future, which can promote the research progress of tumor-targeted drugs. Besides, several recent studies have identified CTHRC1 as an effectual prognostic biomarker for predicting tumor recurrence or metastasis [63, 138]. The prediction model built with CTHRC1 can be used as a more accurate prognostic indicator [2, 139].

On the one hand, CTHRC1 changes the adhesion between cells by regulating the expression of molecules such as integrin *β* and MMPs, thereby enhancing cell mobility and promoting tumor metastasis and invasion. On the other hand, CTHRC1 can also stimulate tumor progression and angiogenesis by increasing the expression of some molecules such as TGF-*β*, HIF-*α*, and Ang-2. It is worth noting that CTHRC1 has different cellular localization and mechanisms of action in different cells and different microenvironments. For instance, CTHRC1 is highly expressed in invasive melanoma but rarely expressed in noninvasive melanoma [11]. CTHRC1 has no effect on cell proliferation in cervical cancer and endometrial cancer, but it is necessary for the proliferation of ESCC [2, 50, 55]. CTHRC1 regulates the expression of Ang-2 and promotes the angiogenesis of pancreatic tumors [54], while CTHRC1 secreted by osteoclasts may induce the development of cancerous bone lesions without mediating angiogenesis [90]. This complex network of interactions inextricably links to the malignant progression of tumors. Consequently, research on the mechanism of CTHRC1 activation (or inactivation) and crosstalk of known signaling pathways will promote targeted drug development in the future.

## Figures and Tables

**Table 1:** CTHRC1 regulates multiple signaling pathways to promote tumor development and metastasis.

Disease model/cancer	Related pathway	The effect of CTHRC1 on the signal	Function	Refs
Breast cancer	Wnt/*β*-catenin	+	Proliferation, invasion, migration	[33]
	TGF-*β*	+	Bone lesions	[90]
Ovarian cancer	Integrin *β*3-FAK	+	Invasion, migration	[112]
	PI3/AKT/ERK	+	Invasion, migration	[130]
EOC	WNT/*β*-catenin	+	Invasion, migration	[88]
Endometrial cancer	Integrin *β*3/PI3K/AKT	+	TAM infiltration, invasion, migration	[55]
Urothelial carcinoma	Wnt/PCP	+	Invasion, migration	[99]
NSCLC	WntT/*β*-catenin	+	Invasion, migration	[27]
	FAK/MEK/ERK	+	Invasion, migration	[41]
Gastric cancer	HIF-1*α*/CXCR4	+	Invasion, migration	[132]
GIST	Wnt/*β*-catenin	−	Invasion, migration	[93]
	Wnt/PCP	+	Angiogenesis	[101]
CRC	Wnt*/β*-catenin	+	Invasion, migration	[89]
	TGF-*β*	+	Invasion, migration	[63]
	Erk1/2	+	Invasion, migration	[37]
	MAPK/MEK/ERK	+	Invasion, migration	[23]
OSSC	Wnt/*β*-catenin	+	CTHRC1 up	[52]
	Wnt/PCP	+	Invasion, migration	
Pancreatic cancer	Wnt/*β*-catenin	+	Adhesion, migration	[100]
	Wnt/PCP	+	Angiogenesis	[54]
	Src-FAK	+	Migration	[77]
ESCC	MEK/ERK	+	Proliferation, invasion, migration	[50]
Hepatocellular carcinoma	PI3K/AKT/ERK	+	Proliferation, invasion, migration, angiogenesis	[129]

A plus sign stands for activation; a minus represents inhibition.

## Abbreviations

ALP: Alkaline phosphatase
Ang: Angiopoietin
APC: Adenomatous polyposis coli
bFGF: Basic fibroblast growth factor
BMPs: Bone morphogenetic proteins
c-JNK: c-Jun N-terminal kinase
CK1*α*: Casein kinase 1*α*
CRC: Colorectal cancer
CREB: cAMP response element-binding protein
CTHRC1: Collagen triple helix repeat containing-1
CTRPs: C1q/tumor necrosis factor-*α*-related proteins
DPAGT1: Dolichyl-phosphate N-acetylglucosamine-phosphotransferase 1
DVL: Dishevelled
ECs: Endothelial cells
ECM: Extracellular matrix
EGFR: Epidermal growth factor receptor
EM: Endometriosis
EMT: Epithelial-mesenchymal transition
ERK: Extracellular signal-regulated kinase
ESCC: Esophageal squamous cell carcinoma
FAK: Focal adhesion kinase
FRA-1: Fos-related antigen-1
Fzd: Frizzled
GIST: Gastrointestinal stromal tumor
GSK3*β*: Glycogen synthase kinase 3*β*
GTPase: Guanosine triphosphatase
HBV: Hepatitis B virus
HIF-1*α*: Hypoxia-inducible factor 1*α*
HPV: Human papillomavirus
HUVECs: Human umbilical vein endothelial cells
IRCF: Infarct repair cardiac fibroblasts
LEF: Lymphoid enhancer factor
LGR: Leucine-rich repeat-containing G protein-coupled receptor
LncRNA: Long noncoding RNA
LRP: Lipoprotein receptor-related protein
MALAT-1: Metastasis-associated lung adenocarcinoma transcript 1
MAPK/MEK: Mitogen-activated protein kinase
MI: Myocardial infarction
MiRNAs: Microribonucleic acids
MMPs: Matrix metallopeptidases
mTOR: mammalian target of rapamycin
NFATs: Nuclear factor of activated T-cells
NF-*κ*B: Nuclear factor-kappa B
NSCLC: Non-small-cell lung carcinoma
OSCC: Oral squamous cell carcinoma
PCP: Planar cell polarity
PI3K: Phosphatidylinositol 3-kinase
PKB: Protein kinase B
PKC-*δ*: Protein kinase C *δ*
Rac1: Ras-related C3 botulinum toxin substrate 1
ROCK: RhoA/Rho associated kinase
SH2: Src homology 2
SRC: Steroid receptor coactivator
TCF: T-cell factor
TEMs: Tie2-expressing monocytes
TGF-*β*: Transforming growth factor-*β*
TNM: Tumor-node-metastasis
VEGF: Vascular endothelial growth factor
WAIF1: Wnt-activated inhibitory factor 1.
